# Comparing the short and original versions of the Activities-specific Balance Confidence (ABC) scale in older adults

**DOI:** 10.1093/geroni/igab046.2896

**Published:** 2021-12-17

**Authors:** Chad Tiernan, Allon Goldberg

**Affiliations:** 1 University of Michigan-Flint, grand blanc, Michigan, United States; 2 University of Michigan-Flint, Flint, Michigan, United States

## Abstract

Balance confidence assessment in older adults has implications for falls and quality of life. It remains unclear whether the original Activities-specific Balance Confidence (ABC-16) scale or the shortened 6-item (ABC-6) scale is recommended. To further inform the decision-making process of balance confidence tool selection, a secondary analysis of an existing dataset consisting of 77 community-dwelling older adults was performed. ABC-16 and ABC-6 association and agreement, internal consistencies, and relationships with self-rated health (SRH) were assessed. Participants were primarily female (80.5%) between the ages of 60 and 87 years. Results indicated a strong association between the scales [r = .97, p<.001); ICC(2,1) = .80] but limited agreement (95% Limits of Agreement range = 22.1; mean difference of 7.2 points in the direction of the ABC-16). Cronbach’s alphas were .95 (ABC-16) and .89 (ABC-6), suggesting high internal consistency for both scales but possible item redundancy with the ABC-16. Regression model 1 (ABC-6 = primary predictor) explained more of the variance (R2=.36) in SRH compared to model 2 (ABC-16 = primary predictor; R2=.29). Hotelling’s t-test [t(74)=2.4, p=.008] indicated that the correlation coefficient (Multiple R) from the ABC-6 model was significantly higher than the correlation coefficient from the ABC-16 model. In conclusion, despite a high correlation, the two scales did not agree strongly and should not be considered interchangeable. Given that the ABC-16 takes longer to administer, does not relate to SRH as strongly, and could have redundant items, the ABC-6 should be considered for balance confidence assessment in older adults.

